# Information and Communication Technologies to Support Chronic Disease Self-Management: Preconditions for Enhancing the Partnership in Person-Centered Care

**DOI:** 10.2196/jopm.8846

**Published:** 2017-07-07

**Authors:** Sabine Wildevuur, Fleur Thomese, Julie Ferguson, Ab Klink

**Keywords:** person-centered care, chronic disease management, cancer, self-management, partnership, information, communication, technologies

## Abstract

**Objective:**

In order to alleviate the pressure on health care systems exerted by the growing prevalence of chronic diseases, information and communication technologies (ICT) are being introduced to enable self-management of chronic diseases by supporting partnerships between patients and health care professionals. This move towards chronic disease self-management is accompanied by a shift in focus on integrating the patient with his or her perceptions on the chronic disease as a full-fledged partner into the health care system. This new perspective has been described as “person-centered care” (PCC). To date, information and communication technologies only partially build on the principles of PCC. This paper examines the preconditions of ICT to enable a person-centered approach to chronic disease management.

**Methods:**

Using cancer treatment as a case study for ICT-enabled PCC, we conducted a comparative analysis of thirteen scientific studies on interventions presented as ICT-enabled PCC for cancer treatment, to answer the research question: What are the preconditions of ICT-enabled PCC in chronic disease management? Based on the intended and actual outcomes, we distilled in several analytic steps the preconditions of ICT-enabled PCC for chronic disease self-management.

**Results:**

We distinguished four user-related preconditions of ICT-enabled PCC: (shared) decision making, personalized ICT, health-related quality of life, and efficiency.

**Conclusions:**

We argue that these four preconditions together can improve people’s self-management of chronic diseases by strengthening the partnership between the patient and the healthcare professional. Moreover, the study revealed a discrepancy between intended and reported actual outcomes in terms of realizing person-centered care.

## Introduction

Chronic noncommunicable diseases are the leading cause of illness, disability, and mortality, exerting significant pressure on the sustainability of worldwide health care systems [1]. Management of a chronic disease is often a lifetime task for which the patient is responsible on a day-to-day basis. This requires on the one hand “self-management” by the patient, involving active participation of people in their own health care process, and on the other requires helping them and their families to accrue the knowledge, confidence and skills to manage their condition [2].

Successful self-management of a chronic disease allows people to handle their life with some degree of independence despite their medical condition, and to feel healthy despite their limitations [3]. A key characteristic of self-management is a collaborative approach to the care of chronic illness, in which patients and professionals form a partnership focused on the patient [4]. Thus, rather than perceiving health care professionals as experts and patients as subjects that bring little to the table besides their illness, a self-management partnership means that people with chronic conditions become their own principal caregivers, and health care professionals are seen as “consultants” supporting them in this role [2].

Information and Communication Technologies (ICT) are considered an important enabler of such partnerships, as ICT can offer ways to connect chronic patients and their health care providers around the clock and at a distance, contributing, for example, to more self-monitoring and shorter hospital stays [5,6]. Nonetheless, the partnership is often neglected in the design of ICT applications aimed at supporting chronic disease self-management [7,8]. ICT applications for health care purposes are regularly developed for⎯rather than with⎯the intended users [9]. Moreover, ICT applications typically do not take into account the partnership between patients and health care professionals [10], and are focused on only one of these parties rather than considering both [11]. This lack of consideration for both the patient and the health care professional, as well as their partnership, increases the risk that ICT applications are mismatched with user needs, and that the technology ends up lacking meaning in practice for both patients and health care professionals [12]. Thus, while more and more health care-supporting interventions and applications are being designed, it remains unclear whether and how such interventions in fact contribute to better self-management of chronic conditions. This is problematic, because when the promise of ICT-enabled support tools is not realized, not only significant investments in ICT solutions are wasted, but most of all: collaborative partnerships between patients and health care professionals within health and health care are not optimized.

In this paper, we aim to generate understanding of the preconditions toward realizing ICT-enabled approaches to support chronic disease self-management. We opted for the term preconditions as these best describe the necessary⎯but not exclusive⎯characteristics to realizing actual use. Identifying preconditions to ICT-enabled chronic disease self-management is an important step in improving the technology design process toward better support of the partnership between the patient and health care professional. Building on our analysis, we explain how ICT can be better tailored toward self-management of chronic diseases, for both patients and health care professionals. We draw on the concept of person-centered care (PCC) to guide this analysis, whereby a patient’s personal context and situation informs and guides the design and implementation of their health care. Our case study, based on an analysis of thirteen studies in which ICT was presented as an important means to support person-centered chronic disease management of cancer, is therefore guided by the research question: What are the preconditions of ICT-enabled PCC in chronic disease management?

We identified four preconditions for ICT-enabled person-centered care, but found that while these preconditions are sometimes met, the intended outcomes of ICT-enabled person-centered care are not always realized. We explain this discrepancy by drawing on an affordances perspective, which forefronts the actual use, and not only the designed intent of technology. We first introduce the theory on person-centered care that informed our study.

## Person-Centered Care

Person-centered care (PCC) is a systematic approach to disease management that involves the patient as an equal partner in the care process [13]. Initial studies on person-centered care suggest that a fully implemented PCC approach keeps people more resilient, shortens hospital stays and improves quality of care [14,15]. PCC involves three core components: initiating the partnership, by eliciting a detailed patient narrative; working the partnership between patient and health care professional, by implementing the narratives in the care process through shared decision making; and safeguarding the partnership, by documenting the partnership in the patient record [13]. The patient narrative is the person’s personal account of his illness and symptoms, and their impact on his life. It captures the person’s suffering in an everyday context, in contrast to medical narratives that reflect the process of diagnosing and treating the disease [13]. The PCC components build on each other, and can be reiterated.

PCC can be considered a specific type of shared-decision making, which involves an interaction process established in the partnership between patient and health care professionals [7,15]. Through the combination of this process orientation with a narrative orientation, PCC emphasizes the need to build partnerships based on the personal, individual meaning that a (chronic) disease has in a person’s life. As this is a highly personalized process, ICT applications have the potential through their flexibility to be particularly suitable for supporting these partnerships [6]. Yet, the development of such ICT support for PCC is still in its infancy [7,8]. Our study seeks to further develop this understanding by way of a case study that we now introduce.

## Methods

### Setting and Sample

Our dataset consisted of thirteen cases (listed in Multimedia Appendix 1) derived from a prior large scoping review of literature on ICT interventions in a wide variety of self-management and connected-care activities [8], which presented ICT-enabled health care as an important means to support person-centered chronic disease management. The studies we selected for our analysis followed what could be considered as ICT-enabled person-centered care for chronic conditions, meaning the ICT-interventions were aimed at meeting the three established components of person-centered care: Initiating the partnership (patient narratives); working the partnership (shared decision making) and safeguarding the partnership (documenting the narrative) [13]. We focus on a single chronic condition⎯cancer care⎯as a means for comparison across studies. By focusing on one chronic condition we were better able to compare across studies. Cancer is one of the main types of non-communicable chronic diseases and the condition is a leading cause of disease worldwide. The sample of cancer yielded the largest category within the scoping review of the “big five” chronic conditions (diabetes mellitus, cardiovascular disease, chronic respiratory disease, cancer, and stroke) studied. Moreover, ICT interventions to support cancer care cover a wide variety of self-management and connected-care activities and are, in that sense, a good example of ICT-enabled PCC toward chronic disease management [16,17].

### Study Design

**Figure 1:**
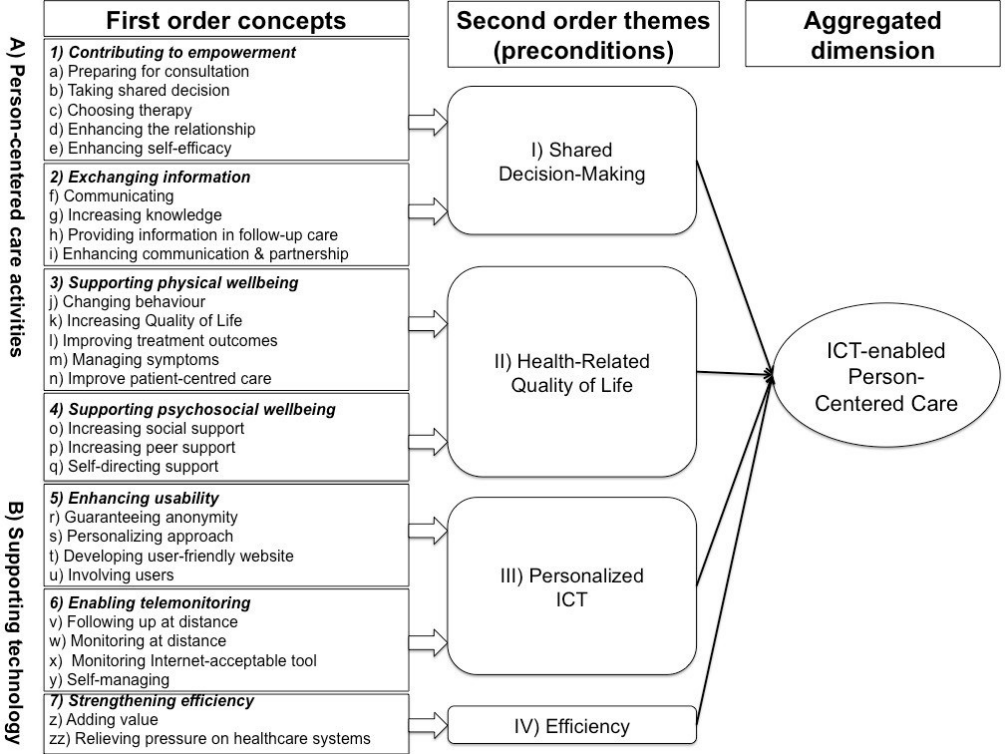
Data structure of ICT enabling PCC.

We analyzed thirteen cases of cancer care by following the initial steps for structuring qualitative data in new concept and theory development, as described by Gioia [18]. The Gioia methodology is a systematic approach using interpretative coding, which was useful for our aim of distilling the preconditions of ICT-enabled PCC based on evidence derived from the selected cases. First, initial (open) coding was conducted in each of the thirteen studies, using NVivo software, whereby we particularly sought to identify how ICT usage was described as a support of chronic disease management in a person-centered approach to care. Second, the first author’s coding was reviewed by the other authors, after which the group of authors grouped them according to similarities and differences in ICT-enabled person-centered care. We created categories by seeking similarities among the codes, grouping these under so-called first-order concepts (summarized in Figure 1), and discussing and adapting these to ensure these first-order concepts were appropriately captured. We looked for patterns among the core concepts, distilling how the described ICT interventions supported disease self-management of cancer in a person-centered approach to care. Third, we identified theoretically-supported second-order themes (“preconditions”) that emerged from the first-order concepts. In the preconditions we articulated the outcomes of the first-order concepts in the interventions studied that afforded a person-centered approach to care, enabled by ICT. The resulting data structure is shown in Figure 1.

We based our preconditions on the ICT-interventions mentioned in the studies. However, not all intended outcomes described were realized. To distinguish intended versus actual outcomes in terms of PCC, we reverted to the originally selected text segments in the cases we studied (summarized in Table 1). These categories were used to recognize if the ICT-interventions enabled person-centered care in chronic disease management not only in theory, but also in health care practice.

## Results

We derived seven so-called “first-order concepts” related to ICT-enabled PCC: contributing to empowerment; exchanging information; supporting physical wellbeing; supporting psychosocial wellbeing; enhancing usability; enabling telemonitoring; and strengthening efficiency (Figure 1). These first order concepts can be seen to represent on the one hand person-centered-care-related activities (A) and on the other the supporting technology (B).

Regarding the person-centered care activities (A), we first identified activities contributing to empowerment (1) that engage patients to “make active choices in their recovery” such as electronic support groups for breast carcinoma [19] These activities were manifested in the form of: preparing for the consultation (1a), taking shared decisions (1b), choosing therapy (1c), enhancing the relationship between the patient and the health care professional (1d) or enhancing self-efficacy (1e). For instance, patient empowerment was mentioned in four studies as being the result of “info-decisional empowerment” (information provision to support decision making), sharing information, and interactive health communication [20,23].

The second first order concept we identified was exchanging information (2), which involves staying in touch outside of regular scheduled sessions, not only with health care professionals but also with supporting peers [19] Exchanging information was manifested through communicating (2f), increasing knowledge (2g), providing information in followup care (2h) and enhancing communication and partnership (2i). Articles describing these activities suggested that ICT increased the opportunities for accessing and exchanging information (eg, [23,17]), as described in the study on the development of a useful, user-friendly website for cancer patient followup by Bartlett and colleagues [17]: “Use of the internet for information exchange between patients and health care staff may provide us a useful adjunct or alternative to traditional followup.”

Supporting physical wellbeing (3) is the third first order concept we distinguished, and involves striving to be as healthy as possible despite the disease [3]. This was manifested in the form of changing behavior (3j), increasing quality of life (3k), improving treatment outcomes (3l), managing symptoms (3m) and improving patient-centered care (3n). For instance, physical well-being, either through behavior change or management of symptoms or treatment, was one of the desired outcomes either through a telephone-based physical activity intervention [24], an online support group for prostate cancer survivors [25], an eHealth application for personalized illness management support [26], a telemedicine system supporting head and neck cancer patients,c and symptom telemonitoring in advanced lung cancer [27]. All cases aimed to have an impact on health-related quality of life. For example, telemedicine systems supporting head and neck cancer patients during the postoperative period at home were beneficial for the quality of life of this group of cancer patients and added to the physical wellbeing of the patients [16].

Next, supporting psychosocial wellbeing (4) involves increasing psychosocial support from being connected to others, for example through a novel patient community. For instance, patients who used an Internet-based, interactive, integrated support system for cancer patients experienced greater social support during the intervention period [28]. Social media also played an important role in psychosocial wellbeing, in particular the use of Twitter as described by Sugawara et al [23]. due to its ability to promote direct interaction between cancer patients.

We also found references to the supporting technology (B), and how it supported cancer self-management in a person-centered manner. First, we identified technology related to enhancing usability (5), which involves the ease of use or the learnability of the ICT applications. One of the few studies that suggested user-involvement in the development process as a means to strengthen usability was Bartlett and colleagues’ [17] analysis, whereby the authors suggest that: “Involving users at developmental stages of eHealth systems is generally considered good practice and can ensure the application under development is both user-friendly and perceived as useful.” Within the cases, usability was represented by guaranteeing anonymity desired by the patients (5r), personalizing approach (5s), developing user-friendly website (5t) and involving users (5u). For example, one of the studies focused on the usability, feasibility and acceptability of a user-friendly and useful website with the potential for use in a “training and website” followup model in cancer care [17].

Enabling telemonitoring (6) in a person-centered approach to care included combining various information technologies for remotely monitoring patients [16,28,29], providing the possibility to following -up at distance (6v), monitoring at distance (6w), monitoring if Internet is an acceptable tool (6x) and self-managing (6y).

Finally, strengthening efficiency (7) involves a substitute for traditional face-to-face followup, which might not be the most (cost-) efficient use of physician and patient time. ICT can offer ways to connect chronic patients and their health care providers around the clock and at a distance. For both the patient and the health care providers the substitute of ICT should be efficient and adding value (7z). Efficiency was sometimes mentioned under the umbrella term “relieving the pressure on health care systems” (7zz). Here, ICT was used for followup at a distance replacing followup visits. This is efficient for both patient and health care professional but is also a means to reduce the pressure on the health care system, including the health care professionals [17].

### Preconditions of ICT Enabling Person-Centered Care

In our third analytical step we developed so-called second order themes based on an iterative analysis between our empirical findings and the literature on person-centered care. We identified four second order themes or “preconditions” of ICT as enabling person-centered care: shared decision making; health-related quality of life; personalized ICT; and efficiency (as summarized in Figure 1).

First, our analysis revealed that shared decision making was a prominent aim in ICT-enabled person-centered care. Shared decision making entails developing the health professionals’ skills in involving patients in decisions related to their treatment, with the aim of increasing the patient’s role in implementing this treatment, and ultimately improving decision quality [30]. ICT supported shared decision making by enabling patients to access online information and thereby gain additional knowledge and a better understanding of their illness, ultimately supporting shared treatment decisions. For instance, Izquierdo et al [31] show how a breast cancer Patient Decision Aid (PDA) allowed patients to adopt a more active role in the choice of treatment options in accordance with their medical and personal preferences.

Second, health-related quality of life consists of both physical and psychosocial wellbeing, which were important first-order concepts in the studies we analyzed. For example, health-related quality of life was mentioned as an outcome of the use of ICT, realized for example through telemedicine, in supporting patients during followup, and resulting in the perceived improvement of symptom control [16]. This was also realized through the use of social media and websites to enable peer support, resulting in an increase in psychosocial wellbeing [23]. The aim of an online self-help support for breast cancer patients was: “We hypothesized that breast cancer bulletin boards would prove to be effective in improving participant’s quality of life as measured by a decrease in depression, and increase in psychosocial well-being and an increase in personal growth.” [18]

We identified personalized ICT as a technology-oriented precondition of ICT-enabled PCC. This was manifested, for instance, through distance monitoring and followup in support of chronic disease self-management, where the capacity for personalized ICT-interventions was recognized as a means to accommodate different needs among patients [17] indicate: “Differences were found between breast and prostate cancer patients and between patients with a first time diagnosis and metastases or recurrences. The large variations among patients in their use of WebChoice components demonstrate that patients’ needs for support vary.” [32]. We also found that peer-to-peer contact was particularly salient as a form of personalized ICT, in that online health communities afforded social support according to personal needs and preferences [20].

Finally, the precondition efficiency arises from the assessment of how ICT could be efficient for both the patient and the health care professional (7z) or to relieve pressure on the health care system (7zz). However, some studies demonstrated concerns that aiming for efficiency through ICT might replace human contact, rather than supporting regular health care efforts. That is, a one-sided emphasis on efficiency through ICT can weaken the partnership between patient and health care professional. For instance, the intended outcome of the ICT intervention of one of the studies was to develop a useful, user-friendly website for cancer patient followup and the site was tested on usability, feasibility and acceptability [17]. Its aim was to use the Internet for followup at a distance between patients and health care staff as a useful adjunct or alternative to traditional face-to-face contact for persons with a low risk of recurrence and with a low level of need. However, the study was initiated to address the burden imposed on health care systems by the growing amounts of followup visits, which put pressure on the workforce of health care professionals. Remote monitoring was proposed as a way to diminish this pressure and decrease the costs, and considered as a low-cost solution to encourage patient self-management. It turned out that patients indicated they wanted to have a way of contacting their health care team without “causing hassle”. However, this was “out with the scope of this study” [17]. Even though the patients were heard through focus groups and interviews, the intervention did not offer the services they wished for with their clinical team. Despite the fact that the authors of the study stated that user involvement in website design can ensure that patients’ needs are met, the expressed wish of the patients for a “personalized” website was not realized. Thus, the intended use of personalized ICT was not the actual outcome.

### Person-Centered Care: Technology in Use

As a final analytical step we sought to understand whether the preconditions we identified actually afforded ICT-enabled person centered care in the studies we analyzed. We compared the described intended use to the reported actual outcomes (“affordances”) of the studies on ICT interventions in practice.

We identified three categories describing whether these routines were actually realized. The first category contained studies that did not report the actual outcome, for example when this was not part of the study design. The second category contained studies whereby the reported actual outcome was equal to the described intended use. The third category comprised studies whereby the reported actual outcome differed from the intended use. The described intended use and the reported actual outcomes are summarized in Table 1.

**Table 1:** Described intended use versus reported actual outcome.

Study		Described intended use	Reported actual outcomes
**Care related**			
	Barlett et al (2012)	Provide information for follow-up care	No actual outcomes reported (only intended use)
		Follow-up at distance	
		Replace face-to-face contact	
		Self-management	
	Gustafson et al (2008)	Regain competence	Enabled social support
		Increase health competence	Increased interactive support
		Empower decision-making	Increased quality of life
		Speed recovery	Increased health competence
		Enable social presence	Enabled feelings of relatedness
	Izquierdo et al (2011)	Increase patient knowledge	Increased shared decision-making
		Promote shared decision-making	Achieved realistic expectations of disease
		Support therapy choice	Reduced passivity decision-making
		Empower decision-making	Increased knowledge on illness
	Lieberman et al (2003)	Encourage empowerment patients	Reduced depression
		Reduce loss of hope	Reduced reaction to pain
		Reduce loss of control	Increased social support
		Reduce unwanted loneliness	Enabled anonymity
			Increased contact outside scheduled hours
	Lieberman et al (2005)	Support peers	Increased psycho-social quality of life
		Support self-direction	
		Social support	
	Ligibel et al (2012)	Increase physical activity	Changed behavior
			Increased physical activity
			Reached lifestyle intervention
	Osei et al (2013)	Increase health-related quality of life	No actual outcomes reported (only intended use)
		Support family members	
	Ruland et al (2010)	Prepare for consultation	Managed symptoms
		Document patient care	
	Ruland et al (2013)	Manage symptoms	Reduced symptom distress
		Support clinicians in more patient-centered, illness-oriented consultation	Improved patient-centered care
		Tailor individual patient needs	Supported symptom management
		Manage disease	
		Manage symptoms	
	Seckin et al (2012)	Empower patient	Prepared for consult
		Self-manage care	Supported coping with cancer
			Empowered info-decision support
	Sugawara et al (2012)	Exchange information	Enabled anonymity
			Empowered through tweeting information
			Supported peers (using Twitter)
			Supported psychologically
			Connected users
	van den Brink et al (2007)	Improve quality of life	Increased impact quality of life
		Communicate	Decreased physical complaints
		Support peers	Reduced uncertainty and fear
		Retrieve information	Increased self-efficacy
			Improved symptom control
	Yount et al (2013)	Relieve symptom distress	Failed to demonstrate efficacy
**Technology related**			
	Barlett et al (2012)	Cost-efficient	Wanted to maintain face-to-face contact patient-health care professional (but not reached)
		Release burden on health care system	Trained prescription
		Monitor telehealth	Involved users in development of eHealth interventions (partly reached)
		Develop user-friendly website	Patients wanted “personalized” website with links to the clinical team (not reached)
			Accessed Internet had to do with personal choice and attitude than ability due to costs
			Differentiated factor of age
	Gustafson et al (2008)	Integrate system of services	Integrated system of services more helpful than usual care
	Izquierdo et al (2011)	Facilitate patient/physician decision-making	Increased understanding of disease
			Deepened awareness of other patients’ experiences
			Encouraged shared decision-making
			Improved quality of decisions
	Lieberman et al (2003)	Deliver electronic support groups through Internet	Occurred technological problems
			Worried clinicians that facilitation would be difficult because of lack usual cues
	Lieberman et al (2005)	Validate Internet bulletin boards	Validated first step bulletin boards
	Ligibel et al (2012)	Intervene with telephone-based exercise	Changed behavior test possible
	Ruland et al (2010)	Tailor individuals through computerized assessment	Improved patient-centered care and patient outcomes, including reduced symptom distress and reduced need for symptom management support
	Ruland et al (2013)	Support cancer patients in illness management	Effectively supported by computer tool
	Seckin et al (2012)	Empower patients	Cyber supported patients for knowledge about their illness and treatment
		Manage computer technology-based information on diseases	
	Sugawara et al (2012)	Role Twitter in the life cancer patients	Exchanged information via Twitter
	van den Brink et al (2007)	Tele monitor to bridge gap after discharge	Felt secure
	Yount et al (2013)	Monitor symptoms	Efficiency not shown

A second category contained studies where the reported actual outcome was equal to the intended use described. An example of this category is the study by Van den Brink and colleagues [16], which focuses on the impact on quality of life of a telemedicine system in support of cancer patients. In this case, the partnership was supported. The intervention group was provided with a laptop and access to a telemedicine support system during the first six weeks after discharge. The system offered possibilities for communication, access to information, peer support and monitoring at home. The study reported that the telemedicine system proved to be beneficial for the quality of life of cancer patients [16].

A third category, which we encountered most often, comprised studies where the reported actual outcome differed from the intended use. These studies revealed a discrepancy between what was described as the intended use of ICT to support chronic disease management and how ICT was actually used in practice, in terms of realizing person-centered care. For instance, in a case describing the development of a useful, user-friendly website for cancer patient followup, the study disclosed that the explicit wish of the patients was to have a way of contacting their health care team without “causing hassle.” [17] However, in the actual realized outcome, the focus was primarily on relieving the burden imposed on health care professionals and the health care system in general through the growing amounts of followup visits. Thus, while remote monitoring of persons with a low risk of recurrence and a low level of need was proposed as a low-cost way to diminish this pressure, decrease costs, and support patient self-management, the intervention ultimately did not offer the services and interactions with their clinical team the patients wished for, even though the patients were consulted in the design process. The intended outcome⎯more patient involvement and patient self care⎯was not realized because patients’ wishes were not met in the design and development process of the ICT-intervention.

Across these categories, only a few studies showed a clear focus on the partnership involving both patients and health care professionals. Nonetheless, partnership is a prerequisite following the original definition of person-centered care by Ekman and colleagues, stating that person-centered care is a systematic approach to disease management that involves the patient as an equal partner in the care process [33] An example where this prerequisite was met was the development process of a patient decision aid (PDA), in which both breast cancer patient and health care professional were involved. “The PDA for breast cancer…has succeeded in improving the quality of decisions for specific situations and has encouraged a shared decision making approach in which both patients and health care professionals take on a participative role.” [31] Clearly, inclusion of the partnership remains a challenge that has, yet, to be overcome if the promise of ICT-enabled PCC is to be met.

## Discussion

The resources needed to support chronic diseases are putting increasing pressure on health care systems. To alleviate this pressure, information and communication technologies (ICT) are being introduced to support self-management of chronic diseases. This move towards chronic disease self-management involves integrating the patient as a full-fledged partner, also described as “person-centered care” (PCC). We argued that ICT only partially builds on the principles of PCC [8], and that explicit understanding of the mechanisms supporting the partnership between patients and health care professionals in ICT-enabled person-centered care is lacking. We therefore sought to identify ICT preconditions in support of chronic disease management as a means to better facilitate a person-centered approach to care and the partnership between the patient and the health care professional in particular.

By analyzing studies reporting a person-centered approach to ICT-enabled cancer care we identified four preconditions: shared decision making, personalized ICT, health-related quality of life, and efficiency. Each of the preconditions involves participation of both patient and health care professional, and emphasizes their collaboration in a partnership rather than treating each partner as an isolated entity. Several studies show that the partnership between patient and health care professional is changing [2,4,15,32]. In participatory medicine, for example, patients are encouraged to act as full partners and are valued as such [32]. ICT has the potential to support participatory medicine by equipping, enabling, empowering and engaging patients, thereby creating a more equal partnership between patients and the health professionals and systems that support them [34].

Through our focus on the health care partnership we extend prior studies addressing the use of ICT to support self-management of chronic diseases that attend to either the experiences of the patients or the health care professionals, but not the participation of both [7,8]. Indeed, we argue that upfront inclusion of different stakeholders of care [35,36] is critical toward more successfully developing and eventually integrating ICT interventions in the health sector. Based on these arguments, we propose the preconditions for a person-centered approach to ICT-enabled care to enhance the effectiveness of the care partnership.

In addition to the four preconditions, we found that the intended use of ICT interventions to enable person-centered care often diverged from the actual use. By analyzing both the intended as well as reported actual outcomes, we sought to understand not only what technology was designed for, but also what it engendered in health care practices. To explain this discrepancy, a “technology affordances” lens is appropriate. Technology affordances relate to the possibilities and opportunities that arise from users engaging with the technology, and take into account the resulting potential behavior changes [33]. In other words, sometimes users tend to use ICT applications differently than intended [37,38] which makes it crucial to examine how users actually engage with a technology over time within a particular setting, and how ICT applications are embedded in their daily practices.

A second insight that the affordances perspective has to offer is that people need to engage with ICT applications to make them have impact. The extensive integration of ICT ushers in significant changes to the actual “fabric” of professional engagement [39]. Our analysis confirmed that simply replacing parts of the workflow with ICT-enabled ways of working barely affects practices [36,40], and ICT cannot be simply added on as an afterthought.

The majority of the cases that we studied (Table 1) showed a discrepancy between the intended use and the reported actual outcomes. Either, the reported outcomes differed from the intended use or the outcomes were not reported at all. Our findings suggest that such a mismatch between intended use and reported actual outcomes might be prevented in future by meeting the preconditions for ICT-enabled PCC.

### Limitations and Further Research

For this study we relied on secondary data of a large scoping review that were not collected for the aim of this study, so we may have missed relevant preconditions that were not described in the studies used. However, we only selected studies that were explicitly aimed at describing the outcomes of ICT-enabled PCC interventions. This means that the likelihood of important omissions is small. Nonetheless, case studies aimed at analyzing ICT-enabled PCC in practice would be useful to validate our findings. This would also enable more in-depth analysis of the ways in which the technology is being embedded within the partnership and the wider organization in which the patient and the health care professional participate.

Another limitation of the study is that it comprises a sample of ICT-enabled cancer treatment cases, excluding other chronic diseases. By limiting ourselves to cancer, we may have missed activities that are specific to other chronic diseases. Further research comparing different chronic disease is a useful way to overcome this limitation and extend the validity of our findings.

This study reflects data from thirteen studies. Since not all of them include a complete overview of the demographics, we lack detailed insights of the interactions between the technology used and the demographics of the persons using it. We therefore do not know to what extent certain outcomes are specific to certain groups, such as effects of education level, cultural background, or age on the engagement with ICT applications.

Overall, more knowledge is needed on the actual use of ICT-interventions in practice and how this supports the partnership between patients and health care professionals in particular. Drawing on the basis we provide in this study, a next step is to combine health innovation research with research on the design of technology-enabled health applications (or “eHealth” [32,34,35]) in a person-centered approach, taking into account the context in which technology is being applied, and most importantly, how people using these technologies experience them in relation to their disease self-management.

## Conclusion

The primary aim of this study was to determine the preconditions of ICT-enabled person-centered care to support a self-management partnership between chronic disease patients and health care professionals. By examining ICT as an important means to facilitate a partnership between patients and health care professionals, we contribute to a nascent body of literature on ICT-enabled health care (or eHealth), and to the relatively new field of research that combines person-centered care and ICT [6,7].

We identified four preconditions to ICT-enabled PCC: shared decision making, personalized ICT, health-related quality of life, and efficiency; but also found that intended and actual use of interventions often diverged. The preconditions all involve participation of both patients and health care professionals as partners in the self-management process. This makes ICT-enabled PCC a partnership that can prove fruitful in furthering participatory medicine.

## Appendix

Multimedia Appendix 1 Selected articles on ICT-enabled person-centered care for chronic disease management of cancer.

## Abbreviations

ICT: information and communication technologies
PCC: person-centered care
PDA: patient decision aid
