# Supplementary material for: Information and Communication Technologies to Support Chronic Disease Self-Management: Preconditions for Enhancing the Partnership in Person-Centered Care
Source: J Particip Med. 2017 Jul 7;9(1):e14. doi: 10.2196/jopm.8846 (PMC8080436; doi:10.2196/jopm.8846)
Supplement: Multimedia Appendix 1 [file jopm_v9i1e14_app1.pdf]

Selected articles on ICT-enabled person-centered care for chronic disease management of cancer

- Bartlett YK, Selby DL, Newsham A, et al. Developing a useful, user-friendly website for cancer patient followup: users' perspectives on ease of access and usefulness. Eur J Cancer Care (Engl). 2012 Nov;21(6):747-57.

- Gustafson DH, Hawkins R, McTavish F, Pingree S, Chen WC, Volrathongchai K, Stengle W, Stewart JA, Serlin RC. Internet-based interactive support for cancer patients: are integrated systems better? J Commun. 2008 Jun;58(2):238-257.

- Izquierdo F, Gracia J, Guerra M, Blasco JA, Andradas E. Health technology assessment-based development of a Spanish breast cancer patient decision aid. Int J Technol Assess Health Care. 2011;27(4):363-8.

- Lieberman MA, Golant M, Giese-Davis J, et al. Electronic support groups for breast carcinoma: a clinical trial of effectiveness. Cancer. 2003 Feb 15;97(4):920-5.

- Lieberman MA, Goldstein BA. Goldstein, Self-help on-line: an outcome evaluation of breast cancer bulletin boards. J Health Psychol. 2005 Nov;10(6):855-62.

- Ligibel JA, Meyerhardt J, Pierce JP, et al. Impact of a telephone-based physical activity intervention upon exercise behaviors and fitness in cancer survivors enrolled in a cooperative group setting. Breast Cancer Res Treat. 2012 Feb;132(1):205-13.

- Osei DK, Lee JW, Modest NN, Pothier PK. Effects of an online support group for prostate cancer survivors: a randomized trial. Urologic Nursing 33(3) (2013) 123-33.

- Ruland CM, Holte HH, Røislien J, et al. Effects of a computer-supported interactive tailored patient assessment tool on patient care, symptom distress, and patients' need for symptom management support: a randomized clinical trial. J Am Med Inform Assoc. 2010 Jul-Aug;17(4):403-10.

- Ruland CM, Maffei RM, Børøsund E, Krahn A, Andersen T, Grimsbø GH. Evaluation of different features of an eHealth application for personalized illness management support: cancer patients' use and appraisal of usefulness. Int J Med Inform. 2013 Jul; 82(7):593-603.

- Seckin G. Informational and decisional empowerment in online health support communities: initial psychometric validation of the Cyber Info-Decisional Empowerment Scale (CIDES) and preliminary data from administration of the scale. Support Care Cancer. 2011 Dec;19(12):2057-61.

- Sugawara Y, Narimatsu H, Hozawa A, Shao L, Otani K, Fukao A. Cancer patients on Twitter: a novel patient community on social media. BMC Res Notes. 2012 Dec 27;5:699.

- van den Brink JL, Moorman PW, de Boer MF, et al. Impact on quality of life of a telemedicine system supporting head and neck cancer patients: a controlled trial during the postoperative period at home. J Am Med Inform Assoc. 2007 Mar-Apr;14(2): 198-205.

- Yount SE, Rothrock N, Bass M, Beaumont JL, Pach D, Lad T, Patel J, Corona M, Weiland R, Del Ciello K, Cella D. A randomized trial of weekly symptom telemonitoring in advanced lung cancer. J Pain Symptom Manage. 2014 Jun;47(6): 973-89. doi: 10.1016/j.jpainsymman.2013.07.013.
